# Human ankle joint movements during walking are probably not determined by talar morphology

**DOI:** 10.1038/s41598-022-17984-5

**Published:** 2022-08-16

**Authors:** Peter Wolf, Roman Moor, Arne Lundberg, Christopher Nester, Anton Arndt, Eveline Graf

**Affiliations:** 1 Sensory-Motor Systems (SMS) Lab, Institute of Robotics and Intelligent Systems, ETH Zurich, Zurich, Switzerland; 2 Department of Clinical Science, Intervention and Technology (Clintec), Karolinska Institute, Stockholm, Sweden; 3 School of Allied Health Professions, University of Keele, Keele, UK; 4 The Swedish School of Sport and Health Sciences (GIH), Stockholm, Sweden; 5 Institute of Physiotherapy, School of Health Sciences, Zurich University of Applied Sciences, Winterthur, Switzerland

**Keywords:** Musculoskeletal system, Bone

## Abstract

Knowledge about the orientation of a representative ankle joint axis is limited to studies of tarsal morphology and of quasistatic movements. The aim of our study was therefore to determine the development of the axis orientation during walking. Intracortical bone pins were used to monitor the kinematics of the talus and tibia of five healthy volunteers. The finite helical axis was determined for moving windows of 10% stance phase and its orientation reported if the rotation about the axis was more than 2°. A representative axis for ankle dorsi- and plantarflexion was also estimated based on tarsal morphology. As reported by literature, the morphology-based axis was inclined more medially upwards for dorsiflexion than for plantarflexion. However, when a mean of the finite helical axis orientations was calculated for each walking trial for dorsiflexion (stance phase 15–25%) and for plantarflexion (stance phase 85–95%), the inclination was less medially upwards in dorsiflexion than in plantarflexion in four out of five participants. Thus, it appears that the inclination of a representative ankle joint axis for dynamic loading situations cannot be estimated from either morphology or quasi-static experiments. Future studies assessing muscle activity, ligament behaviour and articulating surfaces may help to identify the determining factors for the orientation of a representative ankle joint axis.

## Introduction

The ankle joint connects the lower leg with the foot. Specifically, the distal parts of fibula and tibia form the mortise in which the trochlea of the talus fits. The lateral and medial profile of the trochlea have been considered to determine the movement of the ankle joint, constrained by the malleoli and the ligaments crossing the ankle. Generally, measurements of specimens (e.g.,^1,2^) and in vivo imaging and subsequent 3D bone reconstruction (e.g.,^3^) have revealed that the lateral profile represents an arc of a circle with a constant radius while the medial profile is better represented by two arcs of two circles with different radii. Therefore, it was concluded that talar motion relative to the tibia happens along an axis with changing orientation, i.e., the ankle joint does not act as a hinge joint with a fixed axis. As anteriorly the medial radius was found to be smaller than that of the lateral circle, it has been postulated that in dorsiflexion (when the anterior part of the trochlea is uppermost in the mortise and in contact with the horizontal distal end of the tibia) the representative ankle joint axis is inclined medially upwards (joint axis passes through the centres of both circles, the centre of the smaller, medial circle is located more cranial than the centre of the larger, lateral circle). Posteriorly, the medial radius was found to be equally or greater than the radius of the lateral circle. Thus, it has been postulated that in plantarflexion, the representative ankle joint axis is inclined medially downwards^1^. This change of the joint axis inclination, i.e., the change of the angle between the axis and the transverse plane, from medially upwards during dorsiflexion towards medially downwards (or at least inclined less medially upwards) during plantarflexion was confirmed in studies moving specimens under load^4,5^ and in studies imaging the rearfoot and lower leg bones of healthy volunteers in different static positions^6,7^. However, the graphical representation of the ankle axis orientations provided by Sheehan^8^ reveals that during an actively executed plantarflexion movement (monitored by dynamic magnetic resonance imaging) the inclination changes from medially downwards towards a horizontal one. In other words, it might be that the inclination of an ankle joint axis in actively executed movements is less determined by the morphology than assumed.

Studies on rear foot movements are fundamental to diagnose pathologies or to treat injuries of the ankle joint. As the talus has no external landmarks onto which skin markers can be placed, common motion tracking methods applied in gait analysis cannot be used to determine talar kinematics. To be still able to apply common gait analysis methods, foot models have been developed which assumed a single (fixed) axis each for the ankle joint and the subtalar joint^9^. Tracking of markers attached to the calcaneus or a shoe and the tibia can then be used to determine the parameters of both joint axes so that the measured movement is optimally reflected. In a study on unloaded foot movements, this optimisation method resulted in a single ankle joint axis for each of the 14 participants whose orientation was within the range of orientations determined on specimens by Inman (1976)^2^. However, the authors of the study themselves questioned the applicability of the optimisation method when large forces are present^9^. Having determined the accuracy of the optimisation method, Lewis et al. also questioned the applicability of this method to determine the orientation of an ankle joint axis in vivo^10^.

To elaborate the kinematics of the rearfoot bones in vivo, Arndt et al. screwed pins into the tibia, talus, and calcaneus. Markers fixed to the pins were tracked during barefoot walking. For the three participants investigated the comparison of the orientation of the ankle joint axis representing either a plantar or a dorsiflexion movement showed three different characteristics^11^. This inconsistent result is supposedly because very different periods of the stance phase were considered for each participant to calculate the representative axis: A point in time corresponding to the orientation of the reference position and the maximum plantar (or dorsiflexion) were used which were not consistent between participants. Consequently, any comparison of joint axis orientations between the participants is hampered, if not impossible.

This initial work with rear foot bone pins was continued by an international research consortium elaborating barefoot walking^12,13^, walking with different shoes or insoles^14,15^, and slow running^16^. With pins inserted, some participants reduced their gait speed. The participant who reduced his gait speed the most (about 0.1 m/s, equal to less than 10%) also reduced his maximal ground reaction force by about 60 N (less than 10%) while the typical pattern of the ground reaction forces was less “dynamic” but principally preserved. Other changes in gait variables, e.g., angular displacement or plantar pressure distribution, were not systematic and can probably be explained by the general variability of walking rather than by the insertion of the pins^17^. In the very last data acquisition of the consortium, imaging of the bones by computer tomography was also done. Thus, a unique data set on five healthy male participants was gained on bone kinematics and on three dimensionally reconstructed bone surfaces enabling us to elaborate whether an ankle joint axis orientation representative for walking (or parts of the stance phase) can be derived from an axis orientation determined by talar morphology.

As in walking the talus considerably moves relative to the tibia in the sagittal plane, i.e., dorsiflexion and plantarflexion are present in the ankle joint, we were particularly interested in whether in walking the inclination of a representative ankle joint axis also changed from medially upwards during dorsiflexion towards medially downwards in plantarflexion as postulated from morphology studies, and as observed in vitro and in static in vivo measurements.

In contrast to the inclination of an ankle joint axis, no distinct change of the deviation, i.e., the angle between the frontal plane and the axis projected onto the transversal plane, has been reported for dorsi-/plantarflexion, neither based on morphological measurements^3^ nor in static, in vivo postures^7^ nor by movements in vivo without load^8^. Thus, we expected that in walking the deviation of an ankle joint axis did not remarkably change from dorsiflexion to plantarflexion.

## Results

### Walking characteristics

**Figure 1:**
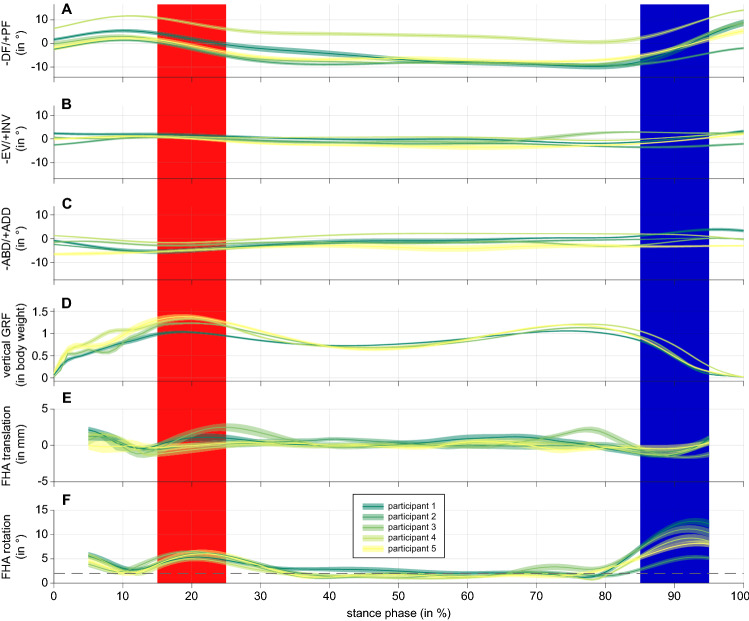
(**A**) Talar position relative to tibia during stance phase of walking in the sagittal plane. Zero degree corresponds to position in relaxed standing. Negative values represent a dorsiflexed (DF) position of the talus relative to tibia, positive values a plantarflexed (PF) position. Mean values (straight line) and corresponding 95% confidence interval (shaded area) are presented for the five participants. Red area indicates the period of the stance phase considered as dorsiflexion movement, blue area as plantarflexion movement. (**B**) Talar position relative to tibia during the stance phase in the frontal plane. Negative values represent an everted (EV), positive an inverted (INV) position. (**C**) Talar position relative to tibia during the stance phase in the transverse plane. Negative values represent an abducted (ABD), positive an adducted (ADD) position. (**D**) Vertical ground reaction Force (GRF) during the stance phase of walking, scaled to body weight. Participant 2 not shown as data acquisition failed. (**E**) Translation along the finite helical axis. As for the calculation of the finite helical axis two time points were chosen being 10% of stance phase apart, values are presented from 5 to 95% stance phase. (**F**) Rotation about the finite helical axis. The orientation of a determined finite helical axis was subsequently only considered when a minimum of 2 degrees (dashed line) was present.

**Figure 2:**
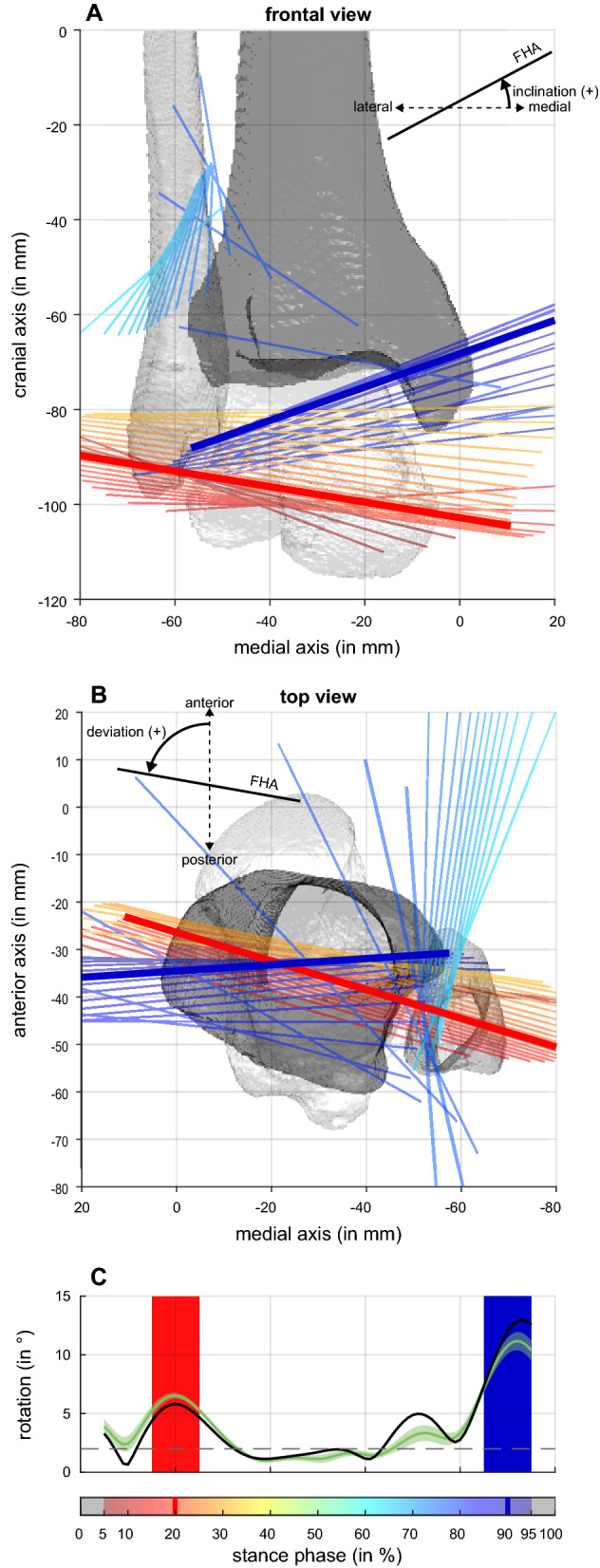
(**A**) Frontal view and (**B**) top view on ankle joint axes observed for an exemplary walking trial of Participant 3. Bones of the right lower limb are displayed in reference (standing position). Joint axes were estimated by finite helical axes calculated for two time points being 10% stance phase apart. Axes were only displayed when rotation about them was greater than 2 degrees (dashed line in **C**). Color code of the axes matches the point in time of the stance they represent (see color bar in **C**). Thicker lines correspond to axes determined for 20% (red) and 90% (blue) stance phase. (**C**) Rotation about finite helical axis shown for the exemplified trial (black line), the mean of all trials of Participant 3 (green line) and 95% confidence interval of the mean (green shaded area). Red area indicates the period of the stance phase considered as dorsiflexion movement, blue area as plantarflexion movement.

Between 13 and 22 trials per participant (all male) could be considered for further analysis. The mean, self-selected gait speed was between 1.3 and 1.5 m/s, with the slowest trial of a participant being at most 12% slower than his fastest. Vertical ground reaction forces typical for walking were observed, with a local minimum in the middle of the stance phase: The vertical ground reaction force decreased compared to the maximum ground reaction force during the phase of weight acceptance, i.e., Fz3 vs. Fz2 as defined by Stacoff et al.^18^, on average by 31 to 54 per cent per participant, and at least by 18 to 43 per cent per participant. Motion in the ankle joint ranged on average between 11 and 19 degrees in the sagittal plane and less than half of that in the other planes (see Fig. 1 and Table 1). In general, the position and orientation of an ankle joint axis determined for moving windows of 10% stance phase changed continuously (exemplified in Fig. 2).

**Table 1:** Personal details of the Participant 1…5 and their walking characteristics.

	Participant 1	Participant 2	Participant 3	Participant 4	Participant 5
**Personal details**					
*Age* (in years)	36	57	35	38	32
*Height* (in m)	1.8	1.83	1.73	1.82	1.8
*Weight* (in kg)	70	94	75	112	71
**Walking characteristics**					
*Number of trials evaluated*	16	22	19	15	13
*Walking speed*: mean (in m/s)	1.34	1.43	1.49	1.51	1.53
& low…upCI95% of mean (in m/s)	1.31…1.37	1.41…1.45	1.47…1.50	1.47…1.55	1.48…1.57
*Stance duration*: mean (in s)	0.659	0.611	0.648	0.641	0.614
& low…upCI95% of mean (in s)	0.651…0.667	0.605…0.618	0.638…0.659	0.622…0.659	0.599…0.628
*Fz2*: mean (in BW)	1.04	n.a	1.24	1.34	1.39
& low…upCI95% of mean (in BW)	1.01…1.07	n.a	1.22…1.27	1.31…1.37	1.34…1.44
*Fz3*: mean (in BW)	0.72	n.a	0.68	0.68	0.64
& low…upCI95% of mean (in BW)	0.70…0.73	n.a	0.67…0.69	0.66…0.70	0.60…0.67
*Fz4*: mean (in BW)	1.06	n.a	1.13	1.21	1.19
& low…upCI95% of mean (in BW)	1.05…1.06	n.a	1.11…1.15	1.20…1.23	1.18…1.21
**Ankle joint kinematics**					
*Sagittal plane ROM*: mean (in °)	19	11.3	17.9	13.6	13.5
& low…upCI95% of mean (in °)	18.0…20.0	10.9…11.8	16.6…19.2	12.5…14.7	12.6…14.4
*Frontal plane ROM*: mean (in °)	5.5	5.2	5.4	3.2	6
& low…upCI95% of mean (in °)	4.9…6.1	4.8…5.6	5.0…5.7	2.7…3.6	5.4…6.6
*Transverse plane ROM*: mean (in °)	9.7	5.6	4.2	4.4	5
& low…upCI95% of mean (in °)	8.2…11.1	5.5…6.2	3.7…4.8	4.0…4.8	4.2…5.7

In addition to mean values, the corresponding lower and upper limit of the 95% confidence interval (low…upCI95% of mean) are reported. For definition of Fz2, Fz3, and Fz4, see Stacoff et al.^18^, all scaled to body weight (BW).

### Ankle joint axis orientation: Derivation from morphology vs. from walking trials

**Figure 3:**
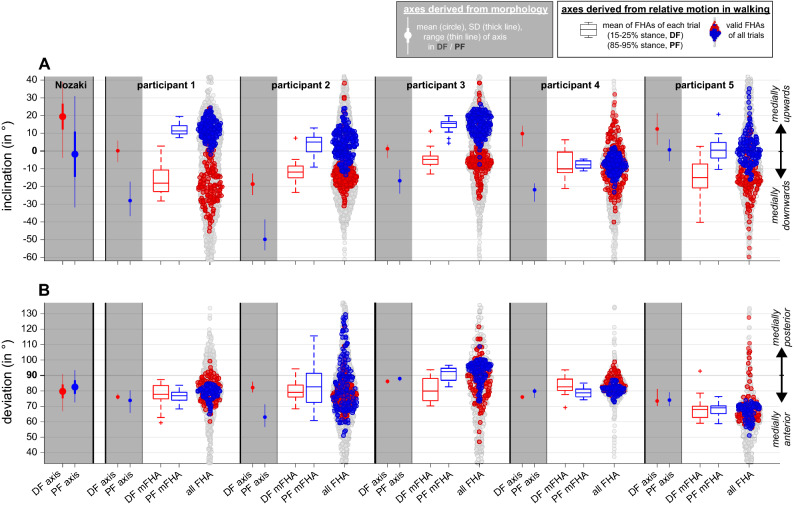
(**A**) Inclination and (**B**) deviation of representative ankle joint axes (for definition of inclination and deviation, see also Fig. 2) derived from morphology (grey background) and from walking trials (white background) for dorsiflexion (DF, red) and plantarflexion (PF, blue). Next to the participants 1 to 5, axes orientations derived from morphology reported by Nozaki et al.^3^ are presented. Boxplots represent mean of finite helical axes (mFHAs) of each walking trial either in dorsi- or plantarflexion. Swarm plots represent orientations of all FHAs with a rotation greater than 2° for the entire stance phase (grey), overlaid by those during DF (red) and by those during PF (blue).

When the ankle joint axis orientation for a dorsiflexion and a plantarflexion movement was estimated from the morphology of the trochlea of the talus for the five participants, the dorsiflexion axis was more medially upwards (or less medially downwards) inclined than the plantarflexion axis, as in^3^. However, when a mean of the finite helical axis (FHA) orientations was calculated for each walking trial for dorsiflexion (stance phase 15–25%) and for plantarflexion (stance phase 85–95%), its inclination was less medially upwards in dorsiflexion than in plantarflexion in four out of five participants (see Fig. 3A and Table 2). The corresponding interquartile ranges did not overlap. When all axial inclinations (around which at least 2° of rotation occurred within 10% of the stance phase) were considered, the corresponding swarm diagrams overlapped only for participant 4 for the phase of dorsiflexion and plantarflexion (see Fig. 3A). Regarding the deviation of the ankle joint axis, such a visual difference was neither found for the comparison of dorsiflexion/plantarflexion nor for the comparison of the derivation from the morphology and the derivation from the walking tests (Fig. 3B).

**Table 2:** Characteristics of an ankle joint axis representative for the dorsiflexion and plantarflexion period in walking.

	Participant 1	Participant 2	Participant 3	Participant 4	Participant 5
**Dorsiflexion period (15–25% stance phase)**					
*Number of valid FHAs* (max. 11 per trail)	175	242	209	165	142
*Plantarflexion per FHA window*: grand mean (in °),	− 4.4	− 5	− 5.7	− 4.1	− 4.6
& min, 5perc, 95perc, max (in °)	− 6.9, − 6.5, − 2.5, − 1.4	− 7.4, − 6.6, − 2.6, − 0.8	− 7.0, − 6.9, − 3.8, − 2.8	− 6.4, − 5.7, − 2.0, − 1.4	− 7.9, − 7.6, − 2.1, − 0.4
*Inversion per FHA window*: grand mean (in °)	− 0.7	− 0.7	− 0.6	− 0.6	− 1.6
& min, 5perc, 95perc, max (in °)	− 2.0, − 1.6, 0.3, 1.0	− 3.0, − 2.2, 1.1, 2.3	− 2.9, − 2.0, 1.7, 2.6	− 3.0, − 1.9, 0.3, 0.5	− 3.0, − 2.8, 0.9, 1.4
*Adduction per FHA window*: grand mean (in °)	1.1	1.1	0.3	0.3	− 1.1
& min, 5perc, 95perc, max (in °)	− 2.5, − 1.3, 2.9, 2.9	− 2.3, − 0.6, 2.2, 2.7	− 4.2, − 2.3, 1.8, 2.6	− 2.0, − 1.6, 1.7, 2.4	− 1.1, − 0.7, 2.6, 2.8
T*ranslation along FHA*: grand mean (in mm)	0.8	− 0.1	1.4	− 0.6	0
& min, 5perc, 95perc, max (in mm)	− 1.0, − 0.8, 2.5, 2.8	− 2.6, − 1.4, 1.8, 2.1	− 2.9, − 2.2, 3.5, 5.1	− 2.4, − 1.9, 0.5, 0.7	− 1.9, − 1.1, 0.9, 1.0
*Rotation about FHA*: grand mean (in °)	4.9	5.5	6	4.5	5.4
& min, 5perc, 95perc, max (in mm)	2.2, 3.2, 6.8, 7.4	2.5, 3.6, 6.9, 7.7	3.3, 4.7, 7.1, 7.3	2.3, 2.9, 6.0, 7.2	2.3, 3.4, 8.1, 8.4
*FHA inclination*: grand mean (in °)	− 15.5	− 11.6	− 4.2	− 6.9	− 14.6
& min, 5perc, 95perc, max (in °)	− 45.0, − 36.3, 11.0, 24.1	− 30.9, − 21.6, 3.6, 38.2	− 26.5, − 19.4, 15.7, 38.3	− 40.0, − 25.8, 16.6, 32.0	− 59.8, − 33.8, 7.2, 18.6
*FHA deviation*: grand mean (in °)	77.6	79.6	80.9	83	69.1
& min, 5perc, 95perc, max (in °)	53.6, 58.4, 91.6, 99.1	51.4, 63.7, 97.7, 138.2	47.2, 68.1, 104.2, 121.4	61.1, 73.9, 92.7, 98.7	55.7, 58.5, 95.7, 127.5
**Plantarflexion period (85–95% stance phase)**					
*Number of valid FHAs* (max. 11 per trail)	176	235	209	165	143
*Plantarflexion per FHA window*: grand mean (in °)	9.9	4	9.2	7.5	6.4
& min, 5perc, 95perc, max (in °)	4.1, 5.5, 13.5, 15.0	1.2, 1.8, 6.2, 6.9	4.1, 5.9, 12.5, 13.5	3.7, 4.5, 10.4, 11.0	2.4, 6.8, 8.6, 8.9
*Inversion per FHA window*: grand mean (in °)	2.6	0.5	− 0.2	1.7	2.3
& min, 5perc, 95perc, max (in °)	0.4, 1.1, 4.6, 5.0	− 2.3, − 1.6, 2.0, 2.6	− 1.5, − 1.3, 1.4, 2.2	0.6, 0.7, 2.7, 2.8	0.9, 1.1, 3.4, 3.6
*Adduction per FHA window*: grand mean (in °)	2.3	0.4	2.3	− 0.9	0
& min, 5perc, 95perc, max (in °)	0.1, 1.0, 3.7, 4.4	− 0.9, − 0.5, 1.3, 1.9	− 0.7, 0.7, 3.4, 3.7	− 2.3, − 2.0, 0.1, 0.2	− 1.6, − 1.4, 2.0, 2.5
*Translation along FHA*: grand mean (in mm)	− 0.8	− 0.4	− 1.3	− 0.8	− 0.2
& min, 5perc, 95perc, max (in mm)	− 2.4, − 2.1, 1.0, 3.6	− 3.8, − 2.8, 1.7, 2.8	− 3.7, − 2.3, 0.1, 1.3	− 2.5, − 1.8, 0.6, 1.1	− 2.0, − 1.8, 0.8, 1.3
*Rotation about FHA*: grand mean (in °),	11.2	4.5	7	8.1	7.2
& min, 5perc, 95perc, max (in mm)	5.4, 7.3, 14.1, 15.2	2.1, 2.5, 6.5, 7.2	5.1, 7.0, 12.7, 13.7	4.5, 5.5, 10.8, 11.4	4.3, 4.8, 9.3, 9.5
*FHA inclination*: grand mean (in °)	12.1	3.9	14.5	− 7.8	1.4
& min, 5perc, 95perc, max (in °)	− 1.9, 3.1, 21.6, 24.5	− 13.4, − 9.0, 16.9, 24.0	− 7.4, − 4.8, 22.8, 26.4	− 23.1, − 15.9, − 0.4, 1.9	− 15.9, − 11.3, 18.0, 35.1
*FHA deviation*: grand mean (in °)	76.3	82.4	91.1	78.8	67.9
& min, 5perc, 95perc, max (in °)	58.1, 67.4, 83.5, 84.8	51.3, 61.4, 116.4, 129.3	73.5, 79.9, 99.2, 108.7	71.4, 73.0, 83.7, 87.0	51.3, 58.3, 75.6, 79.1

### Ankle joint axis orientation in quasi-static vs. dynamic flexion

**Figure 4:**
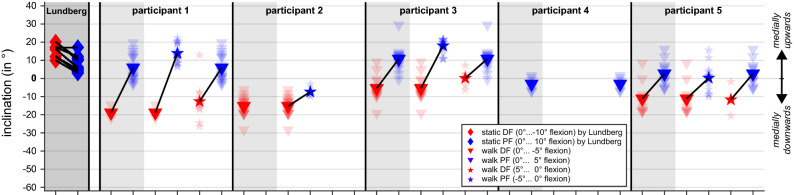
Inclination of ankle joint axes representative for dorsiflexion (red) and plantarflexion (blue). Diamonds represent inclinations of axes which Lundberg et al.^7^ determined for quasi-static flexion in eight participants (black lines link related data). In our study, during the stance phase of walking, the ankle joint was for none of our participants once at least 10° dorsiflexed (i.e., − 10° flexion) and only once at least 10° plantarflexed (i.e., 10° flexion, see Fig. 1). Thus, we report here on joint axes inclinations derived from 5° flexion, either when 5° of dorsi/plantarflexion have been achieved from a joint configuration corresponding to the relaxed standing, i.e., 0° flexion (triangles), or when a joint configuration corresponding to the relaxed standing has been achieved again after a 5° dorsi/plantarflexion position (pentagrams). Inclinations of individual trials, if the axis could be determined, are shown more transparently, mean values per participant and comparison of dorsiflexion and plantar flexion are connected with a black line.

In the study by Lundberg et al.^7^, in which a dorsiflexion and plantarflexion axis in the ankle joint was determined by standing first on a horizontal platform and then in dorsiflexed positions of 10°, 20°, 30° (manually measured by a goniometer between lower leg and fixed horizontal platform) and also in plantarflexed position (by tilting the platform from 0° to 30° of plantar flexion in steps of 10°), the inclination of the representative axis was more medially upwards for dorsiflexion than for plantarflexion in seven of eight participants. In contrast, in the present study, any made comparison of a flexion movement of 5° during stance phase of walking revealed that the finite helical axis of the ankle joint was on average inclined more medially downwards in dorsiflexion than in plantarflexion (see Fig. 4).

## Discussion

A fundamental understanding of the ankle joint kinematics is essential to diagnose and appropriately treat ankle joint injuries or diseases. Only with knowledge of the position and orientation of an ankle joint axis, which represents a movement of interest, it becomes clear how and with which lever arm muscles act on the joint. However, no muscles are attached to the talus. Movements of the talus in relation to the lower leg are therefore caused by movements of neighbouring bones. These movements are transferred to the talus via passive structures such as ligaments or via articulating surfaces. Due to the distinct form of the trochlea, it is obvious that in many studies the morphology of the talus was used to estimate the position and orientation of a representative ankle joint axis. Following the method applied by Nozaki et al.^3^, we also estimated the inclination of an axis for a dorsi- or plantarflexion movement based on the morphology of the talus. We manually selected points on the trochlea to get the relevant arcs required to calculate the axes. This manual selection explains the variation in axis orientation in repeated calculations. Despite this variation, in four out of our five participants all estimated axes were inclined more medially upwards for dorsiflexion than for plantarflexion. The same relation was present for the mean values of the fifth participant. We have thus confirmed numerous comparable studies in which the inclination of an ankle joint axis was also estimated on the basis of morphology (e.g.,^1–3^) and conclude that our participants have a "typical" morphology of the trochlea.

In the sagittal plane, ankle motion was distinctive in all participants during walking accompanied by the greatest range of motion (see Fig. 1 and Table 1). Transverse and frontal plane ankle motion were also considerable which is in line with results already reported in the literature (e.g.^11,19^), especially with those of Lundgren et al.^13^, whereby four of their participants were measured again for the present data collection, at the same laboratory with the same method, simply about 4 years later (participants 2–5 of Lundgren et al.^13^ correspond to participants 2–5 in the present study). Since vertical ground reaction forces of our participants were characterised by an unloading phase in the middle of the stance phase and since we have also shown (on parts of the present data set—at that time, the first set of walking trials with intracortical pins was considered and not those of the second set as well, which were only processed further in the context of the present work) that gait dynamics were not significantly changed using intracortical pins (see^17^), we assume that our kinematic data represent walking of healthy (male) adults.

To derive the orientation of an ankle joint axis during walking, the transformation matrix between two joint positions was calculated according to Woltring et al.^20^. The orientation of the corresponding finite helical axis (FHA) was extracted according to Spoor and Veldpaus^21^. We chose joint positions that were 10% of the stance phase apart (i.e., the orientations could be determined for 5–95% of the stance phase). To minimise the influence of measurement errors, only axes about which a rotation of at least 2° occurred were analysed further. The threshold was chosen empirically, but considered other studies which, for example, chose a threshold of 1° for calculations based on markers directly attached to the skeleton (via the teeth, see^22^) or recommended a threshold of 7° for calculations based on skin markers on the foot^23^ (higher threshold as data from skin markers is noisier). A longer stance phase interval between the two joint positions considered might have resulted in more axes fulfilling the minimal rotation criteria (or allowed a larger threshold to be chosen). However, a longer stance phase interval carries the risk of a reversal of the movement within the time window, so that a representative joint axis can no longer be meaningfully derived. Within the stance phases we selected for dorsi- and plantarflexion, one direction of movement prevailed for all participants and the rotation around the axis of rotation was also clearly above 2° for each moving window (see Fig. 1). A basic comparison of the axis orientations for dorsiflexion and plantarflexion should therefore be valid. Note that the location of the FHA was not within the scope of this study. The location is affected by translation along the FHA, especially when small rotations are present, and should therefore only be analysed in detail after validation of a threshold value for minimal rotation and acceptable translation.

As is usual for studies with only a few participants^24^, we visually analysed the data obtained. Looking at the mean axis positions per dorsi- or plantar flexion phase per walking trial, summarised in boxplots, as well as at all individual axis positions, summarised in swarm plots, reveals that in four out of five participants a representative ankle joint axis was inclined contrary to the estimate from the morphology. Following the work of Lundberg et al.^7^, positions representing a dorsi- or plantar flexion of 5° compared to relaxed standing (i.e., 0° flexion) were also compared—but during the stance phase of walking and not as done by Lundberg et al.^7^, who studied standing on a platform which was either tilted by 10° for plantarflexion or on which participants moved into a dorsiflexion position of 10° between the platform and the lower leg. They did not report the resulting ankle flexion; however, it can be assumed that the ankle flexion was less than 10° at least for dorsiflexion as the reported rotations along the helical axis ranged from 4.2 to 9.6 degrees (as the axis did not coincide with a pure flexion axis, the amount of "projected" flexion was less than the rotation along the axis). Therefore, a comparison to our data with 5° flexion is reasonable. In contrast to Lundberg et al.^7^, in all possible comparisons between 5° dorsiflexion and 5° plantarflexion, we found a more medial upwards inclined axis representing plantarflexion (see Fig. 4). Since movements of 5° were chosen either starting from or resulting in the reference position (i.e., relaxed standing, equal to 0° ankle flexion), similar parts of the central talar dome could be in contact in both movement directions. Thus, it seems that in dynamic cases (and when no extreme joint positions are present) the inclination of the ankle joint axis cannot be estimated from the morphology, at least by the estimations based on morphology suggested so far. In other words, our study revealed FHAs of the ankle joint during walking, and thus information on its intrinsic mechanism, which questions the suitability of foot models based on morphology to estimate ankle joint behaviour in walking.

The change of the inclination of an ankle joint axis representing dorsiflexion to another one representing plantarflexion in walking might therefore be attributed to other aspects than morphology and the change can also not be estimated from quasistatic experiments. Future research assessing muscle activity, ligament behaviour and articulating surfaces will be necessary to better understand the determining factors of an ankle joint axis orientation. For this, active movements could be studied using non-invasive techniques such as dual fluoroscopy^25^, which would remove the invasive nature of our approach and possibly allow more participants to be studied.

Indeed, the small number of participants involved in our experiments is a limitation of our study. However, the more medial upwards inclined axis in "dynamic" plantarflexion compared to dorsiflexion seems to us more given than the opposite prediction based on morphology. In addition, the only comparable work we know of that presented axis inclinations for actively performed movements agrees with our results (cf.^8^, Fig. 3).

From an application point of view, our findings do not have direct implications for the design of artificial ankle joints as long as no single, fixed joint axis is assumed: we have only pointed out that the inclination of the ankle joint axis representing a part of the stance phase changes differently than the morphology would suggest. Differences in axial deviation between dorsi- and plantarflexion—which, as expected from previous studies, were not found—would rather have required a rethinking of how the ankle joint works, as more muscles pass the joint in the transverse plane than in the frontal plane.

## Methods

### Participants

Five healthy men (mean age 38 years, mean weight 85 kg, mean height 181 cm), who were neither prone to foot injuries due to their anatomy nor due to their sport activities and whose last foot injury was at least six months ago, gave informed written consent to participate in our experiments (for personal details, see Table 1). The study procedure was approved by the Stockholm regional ethical committee, Sweden, and the ethical committee of ETH Zurich, Switzerland (EK 2005-N-12). All methods were performed in accordance with the Declaration of Helsinki.

### Study protocol

The study was carried out at the Karolinska Institute, Huddinge, Sweden. In the biomechanical laboratory there, the participants first familiarised with the 10 m walkway. At self-selected speed, participants were asked to hit first a force plate (Kistler, Winterthur, Switzerland, 960 Hz) and then a pressure distribution platform (Novel, Munich, Germany, 50 Hz) with their right foot. Both measurement systems were mounted flush with the walkway and about 1 m apart from each other. Twelve motion capture cameras (Qualisys, Gothenburg, Sweden, 240 Hz) were placed such that kinematics of the right foot from heel strike on the force plate to heel strike on the pressure distribution platform could be tracked. A first measurement session was done with skin-mounted markers followed by a session with intracortical bone pins on the same or next day (The comparison of barefoot walking with pins inserted vs. common barefoot walking has been presented already by Maiwald et al.^17^).

Under sterile conditions and local anaesthesia, two experienced orthopaedic surgeons inserted a self-drilling intracortical bone pin (1.6 mm diameter) into each of the tibia, fibula, talus, calcaneus, navicular, cuboid, medial cuneiform, and metatarsals I and V. After inserting the pins and manually checking for firm pin placement, participants were transported to the biomechanical laboratory. Tripod arrays of 5 mm reflective markers were attached to each of the pins.

The following test conditions were then collected, with two relaxed standing trials recorded prior to each condition to define 0° in the kinematic data: barefoot walking, walking with standard shoe and neutral insole, walking with standard shoe and medially elevated insole, walking with standard shoe and laterally elevated insole, walking with another shoe without midsole modification, walking with a shoe and a first midsole modification, walking with another midsole modification (at least ten trials per condition). After these trials (findings on insole modifications have been presented by Liu et al.^14^, findings on midsole modifications by Arndt et al.^13^), the participants were transported to the in-house radiology department. The right foot was scanned in axial direction from 10 cm proximal to the ankle joint to the sole by a multi-slice computer tomography scanner (LightSpeed VCT; GE Medical Systems, USA). The images (512 × 512 matrix; 0.58 mm × 0.58 mm pixel size, 0.625 mm slice thickness) were acquired in relaxed supine position. After the imaging, participants were brought back to the biomechanical laboratory and another ten barefoot walking trials were recorded again. Subsequently, some participants walked even faster and run slowly. After these trials and about two and a half hours after the start of the pin placement, the pins were drilled out of the bones and the wounds were treated.

### Estimation of the ankle joint axis orientation based on morphology

**Figure 5:**
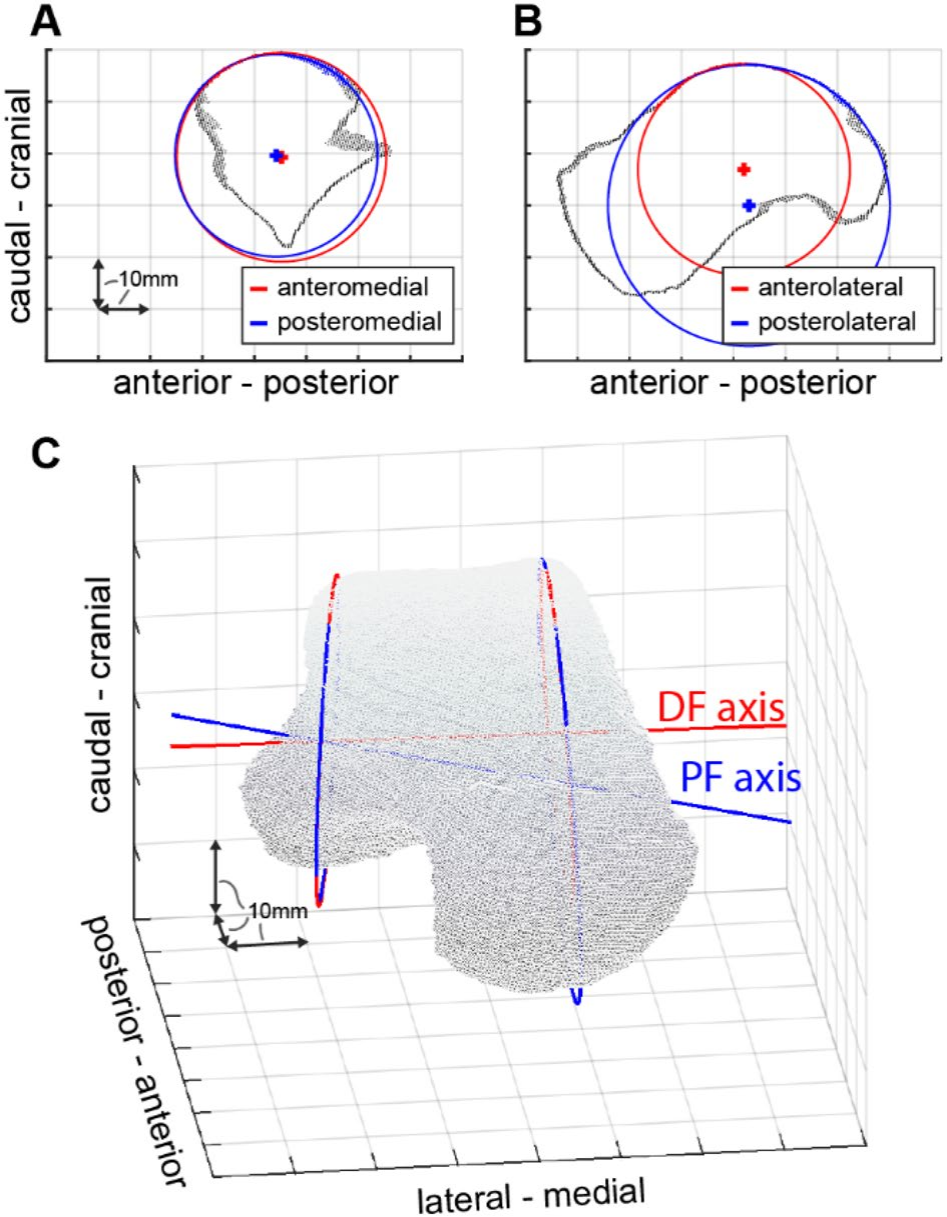
Axis determination based on morphology. (**A**,**B**) Section through the medial and lateral profile of the talar trochlea of Participant 3. Circles were fitted to each anterior and posterior part. (**C**) Corresponding centres were connected and resulted in the axis for dorsi- and plantarflexion. In this example, DF axis was inclined 1.2° and deviated 85.4° whereas PF axis was inclined − 13.2° and deviated 89.4°.

To 3D reconstruct the bones, the images of the feet were semi-automatically segmented by the first author by a commercially available software (AMIRA, v5.2., Mercury Computer Systems, Germany). The segmentation was the same as that used for 3D reconstruction from magnetic resonance images of the foot applied by the first author before, which has been proven to be reliable (see^26^): In the acquisition plane, the appropriate image pixels were assigned to the specific bone and associated markers slice by slice using an intensity threshold function (i.e., the outer border of the bone or marker was detected, and the enclosed area assigned to the bone or marker). Thereafter, the result was controlled in the other two perpendicular planes and if necessary, erroneously segmented areas were corrected. The three-dimensional shapes were exported in virtual reality modelling language format and further processed by custom-written programs in Matlab (MathWorks, USA). The estimation of the axis orientation of the ankle joint representing dorsi- or plantarflexion based on the morphology followed the approach often used in literature: First, both an anterior and a posterior arc of a circle were determined for the lateral and the medial profile of the talar trochlea. The direct connection of corresponding centres was then used to estimate the orientation of the joint axis. Specifically, the talus was first transformed into the relaxed standing position (as the markers arrays were imaged, too). The talus was then visualised, and the first author manually selected three points on the lateral profile of the talar trochlea almost evenly distributed from anterior to posterior. Those points defined a nearly sagittal plane which was also visualised. If the plane did not cut the lateral profile subjectively appropriately, the position and orientation of the plane was corrected by selecting other points until it did. All points closer than 2 mm to the plane were then projected onto the plane and only these points were displayed. This visualisation was then used to manually determine where the trochlea ends anteriorly or posteriorly. Using the most cranial point, two sets of points were created, into each of which a circle was fitted (see Fig. 5, using an approach presented by Taubin^27^).

Starting again from the visualisation of the entire talus, two circles were also determined for the medial profile of the trochlea. The straight line connecting the centres of the anterior circles represented the axis of the ankle joint in dorsiflexion, the line connecting the centres of the posterior circles represented the axis in plantar flexion. Subsequently, deviation and inclination of the axis was determined. The first author repeated the entire procedure five times whereas a dataset of one participant was never reprocessed at the same day. Thus, the ankle joint axis was determined five times each for dorsiflexion and for plantar flexion based on the individual morphological data.

### Determination of the ankle joint axis orientation based on kinematics

All markers were labelled in Qualisys Track Manager and their coordinates exported. A visual check of the coordinates was carried out by the first author for the entire measurement volume in Matlab. Thereby, individual frames that gave the impression of a measurement error (e.g., jump discontinuity) were removed. Marker gaps of up to ten frames were filled by a shape-preserving piecewise cubic interpolation. Marker trajectories were low-pass filtered (4th-order Butterworth filter with cutoff frequency of 10 Hz) and normalised in time to 101 data points, i.e., 0–100%, over the stance phase on the force plate (contact threshold fixed at 10 N vertical ground reaction force). To determine gait speed, the averaged distance of the calcaneal markers between heel strike on the force plate and the next heel strike was used. Since the pressure distribution platform was not synchronised with the force plate and motion capture system, the next heel strike was determined via marker trajectories (based on an approach presented by O’Connor et al.^28^).

A standing trial, which was acquired right before or after a barefoot walking condition, was used as reference pose, i.e., the technical coordinate system of each bone was aligned with the global coordinate system and its origin placed in the centroid of the markers attached to the bone pin. A least square problem was solved as suggested by Söderkvist and Wedin^29^ to get the transformation matrices from the standing trial to each percent of the stance phase.

Theoretically, the three markers fixed to the bone pin formed a rigid body. In practice, vibrations caused by contact with the ground and small inaccuracies of the camera system resulted in small errors in the relative position of the markers. The root mean square of these residual errors was calculated for each percent stance phase. If, within a trial for either the tibia or the talus, the maximum of this error was greater than 1.5 times the interquartile range of all maxima of the participant’s other trials above the upper quartile, the trial was excluded from further analysis (the maximum residual error was usually less than 1 mm).

Rotations in the ankle joint (relative to the standing trial) were then calculated using the helical axis approach^30^: The product of helical axis rotation times the helical axis unit vector was decomposed along the three axes of the coordinate system of the tibia to receive planar joint rotations (presented in Fig. 1A–C).

Two positions of the tibia and talus that were 10% apart in stance phase were considered to calculate a finite helical axis (FHA) of the ankle joint. The orientation of the FHA was then assigned to the middle of the 10% window, i.e., the first axis was estimated for 5% of the stance phase by considering the bone positions at 0% and 10% stance phase. The size of the window was selected to ensure that any movement did happen but no reversal movements. To additionally avoid uncertainties linked to small rotations, only axes about which a rotation greater than 2° happened were further considered. The transformation matrix between two ankle joint positions was calculated according to Woltring et al.^20^, and the orientation of the corresponding FHA was determined according to Spoor and Veltpaus^21^. Inclination was defined as angle between the FHA and the transversal plane, deviation as angle between the projection of the FHA onto the transversal plane and the anterior pointing axis.

### Further data processing and comparison to other studies

Statistical testing was not conducted due to the small sample size. Instead, the determined orientations were visually compared to two representative studies. The first was Nozaki’s work on talar morphology^3^, as several descriptive variables could be extracted from the paper. To compare the morphologically determined axes with those from walking, the range of 15–25% stance phase was defined as dorsiflexion phase and the range of 85–95% stance phase as plantarflexion phase. For each walking trial, a mean value for inclination and deviation was then calculated from the valid finite helical axes (FHAs) for both phases and summarised in boxplots. A second visual comparison was made to Lundberg's results^7^. In that work, the orientation of the ankle joint was determined in quasi-static conditions on eight participants (a platform was inclined by ± 10° about the medial–lateral axis). To allow comparison to our study, we calculated finite helical axes between 0° and 5° ankle flexion, either starting from 0° or from 5°. Both ankle positions had to be either in the interval of 10–30% stance phase or in the interval of 80–100% stance phase (ergo, no fixed interval of 10% stance phase considered, but at most one of 20% stance phase).

## Data Availability

The datasets used and/or analyzed during the current study are available from the corresponding author on reasonable request.
